# MERTK Inhibition as a Targeted Novel Cancer Therapy

**DOI:** 10.3390/ijms25147660

**Published:** 2024-07-12

**Authors:** K.M. Tanim, Alisha Holtzhausen, Aashis Thapa, Justus M. Huelse, Douglas K. Graham, H. Shelton Earp

**Affiliations:** 1 Aflac Cancer and Blood Disorders Center, Children’s Healthcare of Atlanta, Atlanta, GA 30322, USA; ktanim@emory.edu (K.M.T.); aashis.thapa@emory.edu (A.T.); justus.hulse@emory.edu (J.M.H.); 2 Department of Pediatrics, Emory University School of Medicine, Atlanta, GA 30322, USA; 3 Lineburger Comprehensive Cancer Center, University of North Carolina at Chapel Hill, Chapel Hill, NC 27599, USA; alisha_holtzhausen@unc.edu; 4 Department of Pharmacology, School of Medicine, University of North Carolina at Chapel Hill, Chapel Hill, NC 27599, USA

**Keywords:** receptor tyrosine kinase, TAM, MERTK, MERTK Inhibitors, targeted cancer therapy

## Abstract

In this issue honoring the contributions of Greg Lemke, the Earp and Graham lab teams discuss several threads in the discovery, action, signaling, and translational/clinical potential of MERTK, originally called c-mer, a member of the TYRO3, AXL, and MERTK (TAM) family of receptor tyrosine kinases. The 30-year history of the TAM RTK family began slowly as all three members were orphan RTKs without known ligands and/or functions when discovered by three distinct alternate molecular cloning strategies in the pre-genome sequencing era. The pace of understanding their physiologic and pathophysiologic roles has accelerated over the last decade. The activation of ligands bridging externalized phosphatidylserine (PtdSer) has placed these RTKs in a myriad of processes including neurodevelopment, cancer, and autoimmunity. The field is ripe for further advancement and this article hopefully sets the stage for further understanding and therapeutic intervention. Our review will focus on progress made through the collaborations of the Earp and Graham labs over the past 30 years.

## 1. The Early History of MERTK 

### 1.1. The Search for the B-Cell Kinase

The Earp lab began in the era of “second messengers” and intracellular signaling. When the groundbreaking discovery of protein tyrosine phosphorylation by Hunter and colleagues was made [1], the lab turned its attention to the EGF receptor, and in the course of control experiments saw tyrosine-phosphorylated substrates in the membranes of T and B lymphoma cell lines. The T-cell kinase was identified and cloned by Krebs and colleagues several years later and named LCK [2], but the putative B-cell kinase was unknown. The lab exploited the fact that tyrosine kinases evolved in eukaryotes and were absent in bacteria, allowing us to use our homegrown phospho-tyrosine antibodies to detect p-Tyr substrates in lawns of bacteria that had for cloning purposes been transfected with a cDNA library made from a human B lymphoblastoid cell line. Positive colonies by definition must harbor a kinase cDNA fragment that is expressed and functional. The screen identified several kinase sequences eventually cloned by others; our team chose to concentrate on one sequence that was unique. A year of molecular “wrangling” by then-PhD students Doug Graham and Tom Dawson and others in the lab provided a full-length sequence [3,4] and let us know we had identified a transmembrane receptor tyrosine kinase. We had failed to find the “B” cell kinase (eventually identified as Lyn). Examining RNA tissue and cell line expression patterns of the new kinase showed transcripts in cells of monocytic/myeloid, epithelial, and reproductive tissues and led us to the name c-mer. The sequence appeared to be the human orthologue of retroviral oncogene v-eyk, previously identified by the Hanufusa lab [5]; the sequence was also identified independently by the Kung lab [6]. Several years later, geneticists identified the c-mer locus on Chromosome 2 and provided the official name in GenBank as MERTK [7].

In a rather remarkable piece of scientific irony, the lab next door to the Earp lab in the UNC Lineberger building was run by another then-junior faculty member, Edison Liu, who was pursuing a project that he had started while a postdoctoral fellow with Michael Bishop, cloning an unknown transforming oncogene from human CML cell lines. Ed Liu’s approach, led by his then-PhD student John O’Bryan, used the “more” classic oncogene hunting technique of identifying then cloning cDNAs that resulted in transformation of NIH 3T3 cells [8]. They had identified AXL and thus two of the three-membered TAM RTK family characterized by a specific KWIAIES sequence found only in these three kinases, TYRO3, AXL, and MERTK, were identified in adjacent labs in Chapel Hill in the 1990s.

TYRO3 was identified by the person whose career we are honoring with this manuscript, Greg Lemke, whose brilliant idea it was to use the “novel” polymerase chain reaction. He used sequences that were highly conserved flanking the tyrosine kinase domain to seek unique novel tyrosine kinase (TK) domains. He then used those as probes to identify new TKs. His original paper identified 13 new potential kinases [9] and his lab went on to publish the full-length sequence of the third in his list of 12, TYRO3 [10].

To elicit c-mer’s functions and potential role in cancer, Doug Graham studied MERTK in human pediatric leukemia bone marrow samples from the Pediatric Oncology Group. MERTK levels were undetectable in normal resting B and T cells but in clinical samples a significant number of pediatric B-ALL patients had ectopic expression of MERTK and almost half of T-ALL had expression, with the early-stage T-ALL most likely to express [11]. Our collaboration over the years in multiple publications has shown that MERTK is an induced survival factor in leukemia and many solid tumors and is an attractive cancer cell intrinsic therapeutic target reviewed in [12,13].

### 1.2. Characterization of Physiologic Functions

The more physiologic role of MERTK began to be revealed at UNC when working with Bev Koller, who had trained with the pioneer of gene targeting Oliver Smithes, and our group made a *Mertk* knockout (*Mertk*^-/-^) mouse. With Drs Koller and Glenn Matsushima, our graduate student Todd Camenisch made the original *Mertk* knockout (KO) mouse, which developed normally but exhibited a hyperinflammatory phenotype evidenced by a dramatic response to and premature death from low-dose endotoxin [14]. This was the first hint of the myeloid immune phenotype with MERTK as part of the physiologic processes by which the innate immune system can help abrogate chronic inflammation, in essence providing an “innate immune checkpoint” dampening inflammatory cytokine production. We elucidated the mechanism, showing that an antibody to the excess serum TNF-α, detected in the sera of LPS-treated *Mertk*^-/-^ mice, could protect the *Mertk*^-/-^ mice from death. We then showed for the first time that in the absence of MERTK, inhibitory signal NF-κB subunits bound to the TNF-α promoter at higher levels in the macrophages isolated from the *Mertk*^-/-^ mice. The control of cytokine transcription and release is still being worked out, but these experiments pointed the way to mechanisms including regulation of NF-κB promoter binding. The hyperinflammatory state induced by MERTK loss was shown to be even more dramatic in a collaboration with the Lemke lab when his postdoctoral fellow, Dr. Lu, conducted heroic crosses with the UNC *Mertk* KO, Steve Goff’s *Axl* KO, and the Lemke lab *Tyro3* knockout mice [15]. Multiple organs were affected including Sertoli cell function and profound lymphoproliferation was observed.

Next, a seminal observation was made, led by Rona Scott in a collaboration between the Earp and Matsushima labs, the discovery that MERTK was a key component in triggering efferocytosis of apoptotic cells in macrophages [16]. Efferocytosis, as will be discussed below, is triggered in part by MERTK activation by the complex ligand, phosphatidylserine externalized on cells by the process of apoptosis, linked to MERTK (and other TAM RTKs) through the bridging protein ligands GAS6 and PROS1. Anderson and coworkers extended the physiology, showing that PROS1 was the entity in serum that stimulated efferocytosis [17]. Efferocytosis differs from phagocytosis as, for example, in the Scott paper, which demonstrated that impaired apoptotic cell uptake did not show a defect in the phagocytosis of particles or microbes. In addition, efferocytosis is not just confined to myeloid cells, but MERTK appeared to convey some level of this capability on epithelial, endothelial, astrocytes, and multiple cell types (including tumor cells where apoptotic tumor cells can activate MERTK signaling promoting survival and therapeutic resistance). An instructive example of the physiology/pathophysiology of efferocytosis is found in the retina. In the *Mertk*^-/-^ mouse (as well as the RCS rat and rare human families), severe retinitis leading to blindness is due to defective efferocytosis in pigmented epithelial cells [18].

The first hints that the hyperinflammatory phenotype severity of the original *mertk*^-/-^ mouse varies with genetic strain background came from examination of the less severe retinitis pattern in a pure C57/Bl6 mutagenized with ENU leading to a MERTK mutation with less penetrant retinitis [19]. Beautiful mouse genetic, detective work led by Vollrath and colleagues showed the Chromosome 2 location of MERTK and TYRO3 showed differences between the 129 background used to construct the original *mertk*^-/-^ mouse and pure C57/Bl6 *mertk-/-* mouse. Those differences affected TYRO3 expression, with the C57 background expressing up to three-fold higher TYRO3 in the retina, which functioned to ameliorate the severe retinitis phenotype [20]. Another example of the pathophysiology of diminished efferocytosis stimulated MERTK anti-inflammatory action, that we had studied, was defective breast epithelial cell efferocytosis in weaning of the female *Mertk*^-/-^ mouse. This led to inflammatory scarring due to decreased clearance of dying mammary epithelial cells by remaining live epithelial cells during involution resulting from weaning [21].

### 1.3. Identification of Ligands

**Figure 1:**
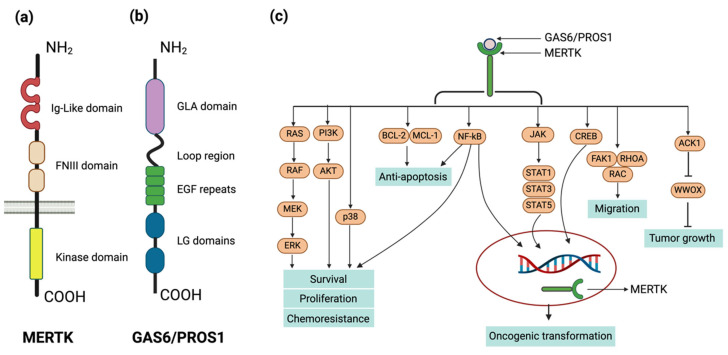
Structure, ligands, and signaling of MERTK. (**a**) Full-length MERTK protein structure. The extracellular domain consists of two immunoglobulin-like domains and two fibronectin type III motifs which facilitate ligand binding and intercellular interactions. The intracellular domain contains the tyrosine kinase domain responsible for signal transduction upon activation. (**b**) Structural domains of MERTK ligands, growth arrest-specific 6 (GAS6) protein, and Protein S. The N-terminus features a gamma-carboxyglutamic acid (GLA) domain, responsible for binding calcium ions and phospholipid membranes, followed by a loop region with four epidermal growth factor-like (EGF-like) repeats that are essential for receptor binding. The C-terminus consists of two laminin G-like (LG) domains that are involved in the binding to TAM receptors. (**c**) MERTK downstream signaling pathways. Events triggered by MERTK receptor following the binding of MERTK’s ligands, Gas6 and Protein S, leading to receptor dimerization and autophosphorylation at specific intracellular tyrosine residues. Subsequent downstream signaling involves the recruitment of various adaptor proteins and the activation of signaling pathways, including PI3K/AKT, MARK/ERK, NF-kB, CREB etc. These pathways contribute to diverse cellular responses including cell survival, cell proliferation, anti-apoptosis, migration.

Several labs undertook the difficult task of isolating protein ligands that bound to TAM RTK family members. Brian Varnum and his team at Amgen who worked with the Liu lab isolated GAS6 as an Axl ligand through large-scale purification of conditioned media [22]. Godowski and colleagues reported GAS6 as a TYRO3/RSE ligand also [23]. Stitt et al. [24] reported that PROS1 was also a ligand. These and multiple other labs have gone on to identify these two bridging protein ligands, whose vitamin K-dependent synthesis produces a Gla-domain that binds to the externalized phosphatidylserine on apoptotic cells through their N terminus and TAM RTKs with their C Terminus. This complex ligand is key to the physiology and pathophysiology of a system design in humans to ingest, silently, billions of dead cells daily, actions that help to prevent chronic inflammation and auto-immunity. Schematics of the MERTK structure and its ligands are shown in Figure 1a,b.

The field is just at the beginning of uncovering the physiologic- and disease-relevant aspects of the TAM RTK family from viruses and leishmaniasis using apoptotic mimicry to their advantage, to roles as biomarkers in lupus and involvement in neurodevelopment and neurodegeneration. The pathophysiology is made more complex by newer data in which mouse model strain differences are showing differences in some phenotypes, but emerging evidence points to tissue as well as context specificities that will make elucidation of TAM RTK action and inhibition even more fun to follow. The articles in this tribute to Greg Lemke and his contributions are setting the stage to understand a ubiquitous process, PtdSer recognition, that is “everywhere you look”.

## 2. MERTK as an Oncogene

While MERTK may not be considered as a classical oncogene with activating gene mutations, multiple studies have supported the concept that overexpression (or ectopic expression) of MERTK can promote malignancy. The convergence of evidence underscores the transformative potential of MERTK and its role in oncogenic processes. As indicated, MERTK expression was not detected in normal resting lymphocytes, yet it was found in both B and T lymphoid leukemia cell lines, as well as in leukemia patient samples, highlighting its aberrant expression in malignancies [11,25,26].

The evolutionary lineage of MERTK reveals its identity with the human ortholog of v-ryk/v-eyk retroviral oncogene found in chickens and was identified through the study of avian retrovirus RPL30. The v-ryk oncogene, a protein tyrosine kinase, was crucial to the RPL30 virus’s ability to induce transformation in host cells and led to a spectrum of cancer types in chickens, as documented by Jia, Hanafusa, and colleagues in 1992 [27].

Beyond its oncogenic implications in avian species, MERTK has been implicated in the activation of several cell survival and proliferation pathways including RAS/MAPK, PI3K/AKT, PLCγ, and JAK/STAT [6,28,29], depicted in Figure 1c. These pathways contribute to MERTK’s cancer-promoting properties, such as its capacity to transform NIH 3T3 fibroblasts [6] and to render Ba/F3 cells independent of IL-3 for survival and growth [30,31].

## 3. MERTK Signaling in Cancer

### 3.1. MERTK Signaling and its Role in AML, ALL, GMB, and NSCLC

MERTK has been identified as having a pathological role in cancer due to its ectopic and aberrant expression across a wide spectrum of human cancers. These range from hematological malignancies, such as leukemia and lymphoma [11,25,32], to various solid tumors such as lung cancer [33], breast cancer [34,35], colorectal cancer [36,37], prostate cancer [38], head and neck squamous cell cancer (HNCC) [39], gastric cancer [40], and glioblastoma multiforme (GBM) [41,42]. This overexpression, in the presence of an abundance of ligand and apoptotic cells in a tumor environment in which there is cell turnover, can contribute to the progression and aggressiveness of the malignancy. For instance, approximately 80–90% of patients diagnosed with acute myeloid leukemia (AML) show elevated levels of MERTK. Similarly, in the case of acute lymphoblastic leukemia (ALL), which affects both children and adults, 30–40% of patients exhibit an overexpression of this kinase [11,25].

The role of MERTK in the development of lymphoblastic leukemia/lymphoma was experimentally tested by constructing a transgenic mouse model that expressed MERTK ectopically in lymphocytes and thymocytes. The overexpression of MERTK resulted in the activation of pathways that prevent cell death, leading to lymphoblastic leukemia/lymphoma in 60% of the MERTK transgenic mice, with hematopoietic expression driven by VAV promoter [43]. MERTK was also aberrantly expressed in mantle cell lymphoma [32]. MERTK and its ligand GAS6 were overexpressed in the bone marrow plasma cells (BMPCs) of multiple myeloma patients compared to healthy controls [44].

In AML cells, targeting MERTK with shRNA in AML cell lines led to decreased MERTK protein levels, resulting in increased myeloblast apoptosis and decreased colony formation. Moreover, mice transplanted with AML cells with MERTK knockdown showed prolonged survival compared to control [25]. Blocking MERTK expression using RNAi increased the apoptotic rate in T-ALL [45]. Inhibition of MERTK in B-ALL prevented ERK1/2 activation, increased the sensitivity of B-ALL cells to cytotoxic agents, promoted apoptosis, and delayed disease onset in a mouse model of leukemia. In B-ALL cell lines, blocking MERTK resulted in reduced activation of AKT and MAPKs, altered gene expression, and enhanced sensitivity to chemotherapy. It also reduced the ability to form colonies and delayed the onset of leukemia in a mouse xenograft model [46].

MERTK overexpression has also been observed in glioblastoma multiforme (GBM). Studies have implicated the overexpression of MERTK in GBM cells as a contributing factor to the tumor’s invasive capabilities and its resistance to programmed cell death, or apoptosis [41,47]. The inhibition of MERTK led to limited migration of GMB and a change in cellular morphology through altered signaling by focal adhesion kinase (FAK) and RhoA GTPase, altering cytoskeletal structure [47]. One of the key mechanisms by which MERTK promotes tumor survival and invasion is through its autophosphorylation in the kinase domain. It activates downstream signaling pathways [48,49] that are crucial for the tumor cells to evade apoptosis, thereby allowing the cancer cells to persist and expand within the brain tissue.

Similarly, in NSCLC, MERTK plays a contributing role in cancer progression. Overexpression of MERTK has been observed in NSCLC tissues and is associated with poor prognosis. This overexpression can lead to the activation of downstream signaling pathways, such as PI3K/AKT, MAPK/ERK, and NF-κB, which promote tumor growth and survival, chemoresistance, and resistance to apoptosis [33,50]. Inhibition of MERTK via RNAi knockdown caused a reduction in NSCLC growth, enhanced chemotherapy sensitivity, and blocked tumor progression in nude mice [33,47,51]. MERTK upregulation has been demonstrated in Osimertinib-resistant NSCLC models. MERTK kinase inhibitor MRX-2843 synergies with third generation EGFR inhibitor Osimertinib to inhibit NSCLC cell expansion, irrespective of EGFR mutational status. These findings provided rationale for an ongoing NSCLC trial evaluating efficacy of combination therapy of MERTK kinase inhibitor MRX-2843 and EGFR kinase inhibitor Osimertinib. Additional clinical trials may explore efficacy of MRX-2843 in KRAS mutant NSCLC [52,53].

### 3.2. Nuclear MERTK

An unexplored, non-canonical signaling mechanism has been suggested by several studies. In acute lymphoblastic leukemia, prolonged GAS6 exposure to MERTK led to the production of a partially N-glycosylated form of MERTK, altering its localization within the nuclear-soluble and chromatin bound fractions [54]. MERTK was observed in the nucleus in dendritic cells (DCs) and had a correlation with the state of dendritic cell differentiation [55]. MERTK localization in the nucleus has also been reported in non-small cell lung cancer (NSCLC). MERTK phosphorylation was required for its nuclear localization in NSCLC [56]. The nuclear localization of TYRO3 and AXL, two other members of the TAM family, has also been documented. Initially, TYRO3 localization was identified in leiomyosarcoma samples [57]. In colorectal cancer, nuclear TYRO3 phosphorylated the epigenetic regulator BRD3 to regulate genes involved in colorectal cancer metastasis [58]. AXL was sequentially cleaved by α- and γ-secretases in cancer cells, which generated an intracellular domain termed as AXL–ICD that translocated to the nucleus [59].

## 4. MERTK: Role in Immune Physiology and Pathophysiology

The early work described in the history section suggested that MERTK (and the other two TAM RTKs) had a role in controlling inflammation. The discovery that MERTK was a key signaling agent in efferocytosis and its absence in knockout mice led to a hyperinflammatory state began to formulate a function for MERTK in the Yin and Yang of immune responses. The interactive innate and adaptive immune system has to function to eliminate foreign entities through a system of recognition, cell alert signals stimulating the adaptive B and T-cell responses. The off signal that prevents injurious chronic inflammation is also critically important.

**Figure 2:**
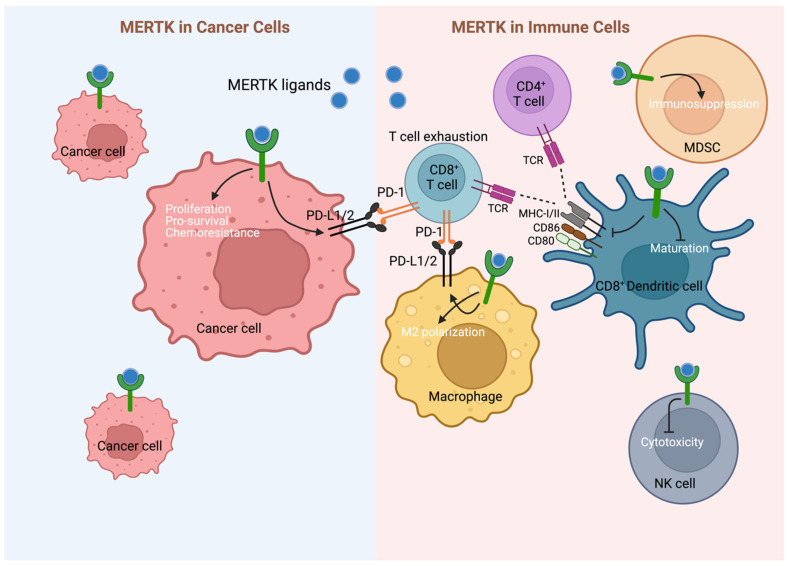
Dual therapeutic effect of targeting MERTK. Inhibition of MERTK may provide direct anti-cancer (LEFT) and immune-mediated (RIGHT) effects. MERTK is frequently upregulated in cancer cells providing pro-survival advantage for cancer cells. Pre-clinical studies demonstrate direct anti-cancer cell effects on MERTK-targeting in cell lines and xenograft models. Targeting the MERTK receptor tyrosine kinases (RTKs) may provide cancer patients with a dual therapeutic effect through direct anti-cancer (**left**) and immune-mediated (**right**) mechanisms. MERTK is frequently upregulated in cancer cells, providing crucial survival advantages, and pre-clinical studies found direct-anti cancer cell effects of MERTK-targeting in cell lines and xenograft models. Activation of MERTK in cancer cells can also induce expression of programmed cell death 1 ligand 1/2 (PD-L1/2), which can suppress anti-cancer immunity by inducing CD8^+^ T cell exhaustion through binding to programmed cell death protein-1 (PD-1). Additionally, MERTK inhibition may boost anti-cancer immunity by overcoming immunosuppressive M2 macrophage polarization and decreasing expression of PD-L1/2 on these cells. MERTK inhibition further promotes anti-cancer immunity by increasing (CD8^+^) dendritic cell (DC) maturation, antigen-presentation capacities, and expression of co-stimulatory proteins, leading to increased anti-cancer CD8^+^ T cell activity, for example in the B cell acute lymphoblastic leukemia (B-ALL) microenvironment. Moreover, MERTK signaling inhibits natural killer (NK) cell cytotoxicity and promotes immunosuppressive functions of myeloid-derived suppressor cells (MDSCs). MHC-I/II, major histocompatibility complex-class I/II; MDSC, myeloid-derived suppressor cell; NK, natural killer; PD-1, programmed cell death-1; PD-L1/2, programmed cell death-ligand 1/2; TCR, T cell receptor.

Two decades of elegant work have identified the T-cell checkpoint mechanism that dampens the T-cell response as important, as are the checks and balances in the innate system that tone down the inflammatory and activation processes. Moreover, the human body must dispose of 5–10 billion dying cells each day and must in some way distinguish the “self” dying cell population lest auto-immunity become rampart. MERTK and its binding of the complex ligand (the Vitamin K-dependent GLA domain containing ligand which binds to exposed PtdSer) which links it to MERTK and triggers ingestion but also regulatory signaling governing the transcription of inflammatory cytokines is a key physiologic signal. The more we learn regarding the exposure of PtdSer on surfaces of patches of cell membranes due to apoptosis or extracellular vesicles or even viruses, the number of processes that MERTK participates in grows. Most of these processes benefit the organism, preventing chronic inflammation or stimulating tissue repair, including wound healing or recovering from myocardial damage. However, the system can be subverted, for example, by viruses or parasites gaining a “foothold” in a myeloid cell population or in the microenvironment in many tumors. Many have contributed to the literature citing instances of TAM RTK function in the tumor microenvironment. MERTK promotes tumor growth by contributing to an immunosuppressive microenvironment (Figure 2). In this section, we will describe several recent papers regarding immunosuppressive infiltrating MERTK+ cells in several syngeneic tumor models.

### 4.1. Immunosuppressive Effects of MERTK in Solid Cancer:

In our initial collaboration, we implanted PyMT-transformed breast cancer tumor cells (which lacked MERTK expression) in the mammary fat pad of wild-type and *Mertk*^-/-^ mice. The tumors demonstrated slower tumor progression and decreased metastasis [60], suggesting that the presence MERTK in the tumor-infiltrating cells, presumably the myeloid population, somehow suppressed an immune response; conversely, absence of MERTK triggered a level of immune tumor rejection, since there was no treatment administered in these experiments. To understand the mechanism, our labs have concentrated on MERTK in myeloid populations, macrophages, myeloid-derived suppressor cells (MDSCs), and dendritic cells.

Eric Ubil initiated a macrophage study [61] and determined that tumor cells express MERTK ligands, GAS6, and Protein S, providing a source of tumor microenvironment (TME) ligand. In B16/F10 melanoma, GAS6 expression increased with tumor growth in vivo while Protein S was expressed at a high level from the onset. Intriguingly, IFN-γ induced PROS1 (similar to IFN-γ induction of T-cell checkpoints). When co-cultured in transwell assay with peritoneal mouse macrophages, the tumor cell secreted PROS1-suppressed inflammatory cytokine transcription induced by a 24-h LPS/IFN-γ treatment of the peritoneal macrophages. When the PROS1 gene was CRISPR-deleted from the B16 cell line, the co-incubation no longer suppressed the M1 cytokines induced by LPS/IFN-γ. When the macrophages were isolated from the peritoneum of the *Mertk*^-/-^ or *Tyro3*^-/-^ mouse, the suppression was also lost, suggesting that PROS1 acting via MERTK inhibits macrophage M1 cytokine production which in the TME would be deleterious to anti-tumor immune stimulation (the suppression was not lost in peritoneal macrophages elicited from the *Axl*^-/-^ mouse). Mechanistically, the authors used several techniques to demonstrate a ligand-dependent MERTK complex formation with PTP1B which sequestered the p38 kinase and inhibited p38-mediated M1 gene expression. In vivo, the wild-type B16 cell line was compared to the same cell line in which PROS1 gene had been CRISPR-deleted in transwell co-culture. Consistent with their in vitro findings, macrophage numbers were increased in B16 tumors derived from the B16 line with PROS1 CRISPR-deleted tumors and there was a decrease in M1 markers without an increase in M2 markers. These changes were accompanied by an increase in tumor-infiltrating CD11b, CD4, and CD8 cells and a decrease in FoxP3 cells, indicating that elimination of tumor-expressed PROS1 changed the immune infiltrate. However, this alone was not enough to affect tumor growth. Rothlin and co-workers had previously demonstrated that TAM RTKs inhibit TLR agonists [62] and the lab tested whether TLR activity can augment PROS1 deletion in the tumor cell. Macrophages treated with resiquimod, a TLR7/8 agonist, and conditioned media from B16F10 cells still exhibited suppressed M1 gene expression; however, when the PROS1-deleted B16 cells were the source of conditioned media, macrophages treated with resiquimod showed restoration of M1 levels. In vivo, when the PROS1-deleted B16 tumors were treated with resiquimod, they showed increased survival, whereas there was no difference in survival in mice bearing B16F10 tumors with or without resiquimod.

Overall, this paper demonstrated a tumor-intrinsic mechanism of immune inhibition applicable to multiple tumor types. This report highlighted several points: the induction of tumor cell PROS 1 by IFN-γ can potentially act as a paracrine source of TME MERTK ligand, a new mechanism of control involving MERTK complexing with the tyrosine phosphatase, PTP1b, and the potential of MERTK action inhibition in lessening the immunosuppressive TME. In this experiment, at least some of the phenotype was achieved in macrophages of *Tyro*^-/-^ mice even though TYRO3 was expressed at a much lower level than MERTK. The TME function of TYRO3 is understudied and far from clarified. The mechanism, complexing with PTP1b, also merits further exploration, although it was validated by showing the loss of PROS1 suppression not only in *Mertk*^-/-^ mouse macrophages but those derived from *Ptp1b*^-/-^ knockout mice.

The TAM RTKs are actively being investigated in other myeloid cell types, for example, Myeloid-derived suppressor cells (MDSCs), which are a potently suppressive immature cell type negatively regulating immune responses in cancer and infection [63]. Recently, Holtzhausen et al. [64] discovered TAM expression in both monocytic MDSCs (M-MDSCs) and polymorphonuclear MDSCs (PMN-MDSCs). Expression of all three TAMs in MDSCs increased in tumor-bearing mice and, consistently, TAM-expressing MDSCs were detected at a higher level in the blood of cancer patients compared to healthy donors. MDSCs exert suppression through a variety of methods, including arginase and iNOS activity, ROS production, and expression of suppressive products like TGF-β and IDO. All of these capabilities were decreased in MDSCs isolated from *Axl*^-/-^, *Mertk*^-/-^, and *Tyro3*^-/-^ mice compared to wild-type (WT). This is in contrast with Ubil’s macrophage work where AXL was irrelevant. In addition, when co-incubated with CD8 T cells, MDSCs from knockout mice suppressed CD8 T cell proliferation much less than WT MDSCs. Interestingly, WT and *Axl*^-/-^ MDSCs were found in both the primary tumor as well as the tumor-draining lymph node (TDLN), but *Mertk*^-/-^ and *Tyro3*^-/-^ MDSCs were predominantly in the primary tumor and not in the TDLN, suggesting that the loss of MERTK or TYRO3 attenuates MDSC migration. In a co-implantation study, tumor cells mixed with *Mertk*^-/-^ MDSCs grew slower than tumor cells mixed with WT MDSCs, indicating that the TME is less suppressive in the absence of TAM RTKs. The authors determined that these suppressive actions are facilitated through a p38–STAT3 signaling axis that is induced by GAS6 but not Protein S. This is also in contrast with Ubil’s macrophage work, which was dependent on PROS1 but not GAS6 [61]. These differences highlight the complexity and context-specificity of TAM RTKs’ functions.

Consistent with data obtained using null MDSCs, when using a pan-TAM inhibitor, UNC4241, suppressive mechanisms were alleviated, and CD8 T cell proliferation was nearly restored in vitro assays. In addition, UNC4241 significantly decreased in vivo BRAF^V600E^PTEN^-/-^ tumor growth and increased CD8 T cell infiltration in vivo. The authors depleted MDSCs, using an anti-Gr-1 monoclonal antibody, implanted tumors, and treated with UNC4241. In the absence of MDSCs, UNC4241 did not suppress tumor growth, suggesting much of UNC4241′s efficacy is exerted on MDSCs. Combination therapy with anti-PD-1 monoclonal antibody treatment further slowed tumor growth and extended survival.

This work demonstrated that TAMs function in a wide variety of cells. It was previously thought that TAM RTKs were not expressed in granulocytic cells; however, they are found in PMN-MDSCs. Also highlighted is that all three receptors play a role in at least mouse MDSC biology, though not necessarily overlapping. In fact, in the analysis of melanoma patients, the level of MERTK+ MDSCs was far higher than those expressing AXL or TYRO3. It is possible that the receptors heterodimerize to be fully active, but this avenue needs more investigation. TAM-expressing MDSCs are found at a higher level in patients with malignancies, opening the door for TAMs to be used as biomarkers and possibly an indicative marker of whether TAM-inhibitors would be an effective therapy.

### 4.2. Immunosuppressive Effects of MERTK in Leukemia

As discussed above, preclinical studies using murine cancer models have implicated MERTK and TAM RTKs in general as promising immunotherapeutic targets in multiple solid cancers. Additionally, recent publications indicate TAM RTKs as potential immunotherapeutic targets in acute leukemia. Host deletion of *Mertk* decreased tumor burden and/or prolonged survival in immunocompetent and syngeneic mouse models of acute myeloid leukemia (AML) and acute lymphoblastic leukemia (ALL) [65,66,67]. Moreover, treatment with the MERTK-selective TKI MRX-2843 recapitulated the extended survival in wild-type (WT) mice in these models. Importantly, anti-leukemia effects were abrogated in immunocompromised mice and the leukemia cells did not express MERTK themselves, indicating that the therapeutic activity was caused by immune-mediated effects and not direct leukemia cell killing [65,66].

Innate immune cells may play an important role in underlying anti-leukemia immune mechanisms, as deletion of *Mertk* selectively in myeloid cells and/or macrophages was sufficient to provide anti-AML immunity and MRX-2843 treatment shifted macrophage polarization toward a pro-inflammatory phenotype in WT mice [66]. Moreover, genetic or pharmacologic MERTK inhibition increased the antigen-presentation capacity of CD8^+^ dendritic cells in a B-ALL model [67] and anti-B-ALL immunity remained partially intact in *Mertk*^-/-^ mice that had severely diminished CD8^+^ T cells, implicating an innate immune mechanism [67]. Indeed, combined depletion of CD8^+^ T cells and CD8^+^ DCs was required for complete abrogation of anti-B-ALL immunity. Similarly, while the optimal anti-AML immune response induced by MRX-2843 was dependent on conventional α/β T cells (comprising CD8^+^ and CD4^+^ T cells), survival was still significantly prolonged in mice lacking these cells [66].

Another important aspect of the underlying immune mechanisms appears to be the regulation of immune checkpoint proteins and T-cell exhaustion. Host *Mertk* knockout or pharmacologic inhibition reduced PD-L1 and PD-L2 expression levels by leukemia-associated macrophages in an AML model [66], and by CD11b^+^ myeloid cells in a B-ALL model [65], while also reducing T-cell exhaustion in these leukemias in vivo and in ex vivo co-culture systems [65,66,67]. Notably, in vitro co-culture experiments with human immune cells and AML cell lines suggested that MERTK inhibition can also induce pro-inflammatory changes in macrophages and decrease T-cell exhaustion in human patients [66].

A recent study found that *Mertk*^-/-^ anti-tumor immunity in melanoma and brain cancer models was not solely mediated by MERTK deletion but required additional genetic changes that had been introduced during generation of the *Mertk*^-/-^ mouse line [68]. In acute leukemia models, however, *Mertk*^-/-^ or pharmacologic MERTK inhibition had similar effects, providing strong evidence that MERTK inhibition is sufficient to elicit anti-tumor immunity [65,66,67]. Since pharmacologic inhibition did not increase survival in immune-deficient mice [65,66], it can be assumed that direct leukemia cell killing did not contribute to therapeutic activity.

In summary, these studies provide rationale for the development of MERTK-targeted anti-leukemia immunotherapies. Notably, the TAM family members TYRO3 and AXL have also been implicated as potential immune-oncologic targets in acute leukemia [67,69], but the role of each individual TAM RTK may be context-dependent: while whole-body knockout of either *Mertk* or *Tyro3* protected against B-ALL [67], knockout of *Axl* only prolonged survival if the kinase was selectively deleted in cells expressing the colony-stimulating factor 1 receptor (Csf1r^+^) [67,69]. Moreover, anti-B-ALL immunity in *Tyro3*^-/-^ mice was less dependent on CD8^+^ DCs compared to *Mertk*^-/-^ mice, indicating a different underlying immune mechanism. Hence, understanding the roles for individual TAM kinases in different cell types and disease contexts may be crucial to determine their relevance as therapeutic targets.

## 5. Potential Therapeutic Effects of MERTK Targeting Agents

**Figure 3:**
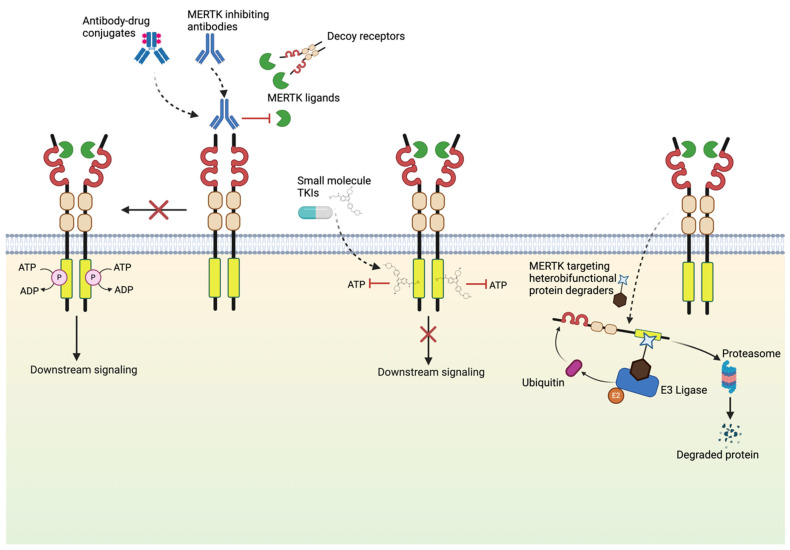
Mechanisms of MERTK inhibition. Multiple MERTK-targeting agents are being developed and refined for use in cancer clinical trials. Potent MERTK-targeting agents in development include selective small-molecule tyrosine kinase inhibitors (TKIs), antagonistic monoclonal antibodies, decoy receptors, antibody–drug conjugates, and heterobifunctional protein degraders that facilitate ubiquitin mediated proteasomal degradation of MERTK.

The above sections point to MERTK (and the other two TAM RTKs) as potential therapeutic targets by at least two mechanisms: (1) Inhibition of these RTKs in the tumor cell itself abrogating growth, survival, and chemo-resistance mechanisms and (2) Acting to reverse the myeloid cell immune-suppressive mechanisms making T-cell checkpoint therapy more efficacious (Figure 3). To that end, several strategies to target MERTK have been developed as MERTK has emerged as an attractive actionable target in a variety of malignancies. Strategies that have proven effective include disruption of ligand–receptor binding with MERTK-specific monoclonal antibodies, ligand sinks, direct inhibition of MERTK kinase activity with ATP-competitive small-molecule inhibitors, and heterobifunctional protein degraders of MERTK, etc. (Figure 3, Table 1 and Table S1).

### 5.1. Biologic Agents

Biologic agents targeting MERTK potentially have higher selectivity towards MERTK resulting in diminished off-target effects compared to small-molecule inhibitors. Mer590, one of the first effective antagonistic monoclonal antibody targeting MERTK, binds to the extracellular domain of MERTK and inhibits signaling by disrupting ligand binding. In an in vitro model of NSCLC, Mer590 was able to reduce cell-surface MERTK levels by 87%, decrease colony formation, increase apoptosis, and increase chemosensitivity to carboplatin [70]. Moreover, Mer590 was also able to reduce migration of a glioblastoma multiforme cell line A172 by 50% [47] and decrease efferocytosis of prostate cancer cell line LNCaP by M2 polarized human monocyte-derived macrophages (hMDM) in vitro [91]. Other antagonistic MERTK antibodies have also been developed that have shown potent anti-tumor effects and enhanced anti-tumor immune response. MERTK antibody Mertk3 in combination with anti-PD1 antibody enhanced tumor growth suppression, promoted anti-tumor immunity, and improved overall survival in a murine model of breast cancer with E0771 cells [34]. In a similar study with E0771 cells, another MERTK antagonistic antibody enhanced anti-tumor effects of anti-PD1 antibody in vivo [71]. In the same study involving a murine model of colorectal cancer (MC38), addition of MERTK antagonistic antibody to the combination of gemcitabine and anti-PD1 led to complete regression of tumors. Taken together, these results demonstrate the potential of antagonistic antibodies against MERTK as monotherapy and part of a combination therapy.

### 5.2. Decoy Receptors

The fact that the extracellular domain of TAM RTK can bind ligands with high specificity and affinity has paved way for development of ligand sinks that can diminish TAM RTK signaling. For instance, MERTK extracellular domain fused to human immunoglobulin G(IgG)-derived Fc domain can act as decoy receptor for GAS6, thus inhibiting GAS6-mediated stimulation of membrane-bound MERTK leading to disruption of MERTK-associated functions in vitro and in vivo [92]. Consequently, recombinant protein dimers comprising of the entirety or part of TAM RTK extracellular domain fused with human IgG-derived Fc domain that can inhibit GAS6-dependent tumor cell survival have been developed. Batiraxcept (AVB-500) is one such recombinant fusion protein with enhanced binding affinity to GAS6 relative to the wild-type AXL sequence. Hence, batiraxcept can inhibit GAS6-mediated AXL signaling [93,94] to exert anti-tumor effects. Notably, batiraxcept monotherapy had a favorable safety profile in a clinical trial for clear cell renal cell carcinoma [95]. Similarly, batiraxcept-based combination therapies have been shown to have favorable safety profiles and promising clinical efficacy in clinical trials for different cancers [95,96,97,98,99]. While batiraxcept might inhibit AXL signaling more potently, it is expected to have inhibitory effect on MERTK and TYRO3 signaling as well, given GAS6 is one of the ligands for both MERTK and TYRO3.

### 5.3. Ligand–Receptor Interaction Inhibitors

A new class of small-molecule inhibitors that target the extracellular domain of TAM receptors to inhibit ligand–receptor interaction is also being developed. Compounds RU-301 and RU-302 are small molecules that bind the interface of the Ig1 AXL ectodomain and GAS6 Lg1 [76]. Based on homology modeling, Kimani et al. predict similar interaction for MERTK and TYRO3. Furthermore, RU-301 and RU-302 disrupted GAS6-inducible TAM activation, inhibited GAS6-inducible cell motility in lung cancer and triple-negative breast cancer cell lines, and suppressed lung cancer growth in a mouse xenograft model [76]. These results provide a potential novel avenue for small molecules to target TAM RTKs in cancers.

### 5.4. Targeted Protein Degraders (TPDs)

Other innovative MERTK inhibitors are also in development. One such class of inhibitors are heterobifunctional degraders, also known as Targeted Protein Degraders (TPD), that target proteins using the ubiquitin proteasome pathway. TPDs might have the added benefit of the ability to counter compensatory increase in MERTK protein levels and stability. Another advantage of TPDs over agents that target the kinase activity of TAM is that TPDs are able to inhibit both kinase and non-kinase activity. KTX-335 (MERTK-selective), KTX-652 (dual MERTK/AXL targeting), and KTX-978 (pan-TAM targeting) are some of the first TAM-selective degraders to be developed that are capable of inducing target degradation as well as functional effects [75]. All three TPDs induced target degradation on reporter cell lines and murine bone marrow-derived macrophages (BMDMs), and inhibited efferocytosis by murine BMDMs ex vivo. Both KTX-652 and KTX-978 reduced proliferation of E0771 murine breast cancer cell line in vitro and induced MERTK degradation in mouse spleen in vivo. Additionally, KTX-978 induced degradation of MERTK in CT-26 tumor (murine colon carcinoma) in vivo.

### 5.5. Small-Molecule Kinase Inhibitors

Although TAM kinases share sequence and structural similarities, a variety of small-molecule inhibitors capable of selectively targeting TAM kinases in single, dual, or pan-TAM fashion are in preclinical and clinical stages of development. Compound 52 was one of the first MERTK inhibitors, albeit a weak one, to be identified in a kinase inhibitor library screen [100,101]. Utilizing the structure-based design approach afforded by the crystal structure of Compound 52-bound MERTK, a series of MERTK inhibitors were developed including UNC569 [77,78,79], UNC2025, and MRX-2843 [102]. The latter two compounds share extensive structural similarities and happen to be the most well-characterized agents in the series, and MRX-2843 has advanced as the clinical candidate (NCT03510104, NCT04762199, NCT04872478, NCT04946890). Studies have demonstrated that these compounds inhibit MERTK phosphorylation and have a variety of functional effects in in vitro cell-based assays and in vivo models of solid tumors and/or leukemia [51,52,65,80,81,82,83,84,85,86,102,103]. Notably, UNC2025 treatment led to reduction in cell density in 30% of 261 primary leukemia patient sample cultures (78 out of 261) as well as in 50% of T-ALL and 40% of AML cultures [80]. A majority of these compounds in this series are dual MERTK and FLT3 inhibitors and generally have selectivity profiles favoring MERTK over other TAM kinases. A variety of other structurally distinct MERTK inhibitors with potent activity in cancer are also available (Table 1). Dual AXL/MERTK inhibitors such as ONO-7475 [104,105,106], INCB081776 [107,108,109], CT413 [87], PF-07265807 [110], and Q702 [111,112], as well as pan-TAM inhibitors like RXDX-106 [40,113], BMS-777607 [114,115,116,117], and LDC1267 [88], have also been found to be effective in cancer models. Moreover, many multi-kinase inhibitors have also be found to be effective in inhibiting MERTK and produce functional effects in cancer models (Table 1 and Table S1). A few such multi-kinase inhibitors, for example, XL092, S49076, sitravatinib, foretinib, and merestinib, have advanced to clinical trials (Table S1).

### 5.6. Agents in Clinical Development

Given the prominent role of MERTK in survival, metastasis, treatment resistance and anti-tumor immunity in a wide range of cancers, MERTK is an attractive target for patients with a spectrum of cancer diagnosis. Indeed, multiple agents targeting MERTK have progressed to clinical trials (Table S1). The first MERTK-selective inhibitor to advance to clinical trials was MRX-2843, a potent dual MERTK and FLT3 inhibitor. A phase I cinical trial of MRX-2843 monotherapy in advanced-stage solid tumors (NCT03510104) was completed recently while another phase I clinical trial of MRX-2843 monotherapy in relapsed/refractory acute leukemias (NCT04872478) is currently underway. Additionally, a phase I/Ib trial of MRX-2843 is currently recruiting to test MRX-2843 in combination with osimertinib (EGFR inhibitor) in EGFR-mutant NSCLC patients (NCT04762199). In addition to MERTK-selective inhibitor MRX-2843, dual selective inhibitors, pan-TAM inhibitors, and multi-kinase inhibitors have also advanced to clinical testing. A phase I clinical trial of ONO-7475 (dual AXL/MERTK inhibitor) with or without nivolumab (anti-PD-1 antibody) in advanced or metastatic solid tumors was recently completed (NCT03730337). Another dual AXL/MERTK inhibitor INCB081776 is also being tested in a phase I study as monotherapy and in combination with retifanlimab (anti-PD-1 antibody) in patients with relapsed/refractory AML or certain advanced-stage solid tumors (NCT03522142). Similarly, Q702, an AXL/MERTK inhibitor, is being tested in early-phase trials as monotherapy (NCT04648254) or in combination with the anti-PD-1 antibody pembrolizumab (NCT05438420).

PF-07265807 (AXL/MERTK inhibitor) is also in an early-phase trial (NCT04458259) as a monotherapy or in combination with sasanlimab (with or without VEGFR TKI axitinib) in advanced-stage solid tumor patients. A phase I study (NCT02791334) with merestinib in combination with the anti-PD-L1 antibody LY3300054 in advanced-stage solid tumor patients demonstrated encouraging anti-tumor effects in some patients [118]. Similarly, favorable safety profile and anti-tumor efficacy with a best response of stable disease in 32% (60 out of 186) of patients was observed in another phase I study (NCT01285037) evaluating merestinib as monotherapy or in combinations involving cetuximab, cisplatin, gemcitabine, and ramucirumab in advanced cancers [119]. S49076, a multi-kinase MET, AXL, and MERTK inhibitor, in combination with bevacizumab was also well tolerated in glioblastoma patients (ISRCTN11619481) in phase I/II study [120]. Another phase I/II study (EudraCT 2015-002646-31) of S49076 in combination with gefitinib also demonstrated tolerable safety profile and limited anti-tumor activity in NSCLC patients [121]. BMS-777607 (ASLAN002), an MET and pan-TAM kinase inhibitor, was also well tolerated in a phase I clinical trial (NCT01721148) involving patients with advanced or metastatic solid tumors [122].

Similarly, a first-in-human phase I/Ib trial (NCT02219711) of another multi-kinase inhibitor sitravatinib demonstrated manageable safety profile and modest clinical activity of sitravatinib monotherapy in solid tumors [123]. Furthermore, a phase I trial (NCT02954991) of sitravatinib in combination with nivolumab demonstrated anti-tumor efficacy and manageable safety profile in patients with advanced-stage NSCLC [124]. The same combination in another early phase I window of opportunity trial (NCT03575598) in oral cavity cancer patients had tolerable safety profile and resulted in deep clinical and pathological response [125]. Moreover, multiple trials are testing sitravatinib in combination with different immune checkpoint inhibitor antibodies and chemotherapy in various cancers (Table S1). Foretinib, a multi-kinase inhibitor capable of inhibiting MERTK, has also demonstrated favorable safety profile and promising anti-tumor efficacy across multiple clinical trials [126,127,128,129]. A summary of relevant therapeutic agents and clinical trials is provided in Table S1.

As current MERTK-targeting agents are further characterized, the potential for translation of clinically relevant MERTK-targeting agents will be clarified. In particular, the exploration of MERTK inhibitors as part of combination therapies may yield new therapeutic options for patients with relapsed or refractory cancers.

## 6. Potential Adverse Effects in MERTK Inhibition

MERTK’s physiological roles in efferocytosis, innate immune regulation, and platelet aggregation may contribute to some potential side effects. MERTK is involved in the phagocytosis of apoptotic cells. Due to the role MERTK plays in clearance of apoptosis in the retina, MERTK inhibition could therefore lead to retinal degeneration and visual impairment [130,131]. In the current clinical trials, vision impairments are being monitored, but none have been observed thus far. Inhibition of MERTK could increase autoantibodies and potentially could increase the risk of inflammation and auto-immune disease [16,132,133]. However, MERTK inhibition alone tends to have a less pronounced effect compared to inhibition of the entire TAM family. In triple knockout mice lacking all three TAM (Tyro3, Axl, Mertk) family receptors, endotoxin-induced inflammation is notable compared to Mertk single knockout mice [14,134]. This provides the rationale that selective targeting of MERTK potentially harnesses therapeutic benefits while minimizing the risk of adverse effects. MERTK is expressed on platelets and enhances blood clot stabilization. Therefore, MERTK inhibition may reduce clot stability. However, in preclinical studies, mice with MERTK knockout or treated with the MERTK kinase inhibitor UNC2025 did not alter bleeding times [135,136]. The risk of these potential side effects of MERTK inhibition could be potentially diminished by changing the duration and timing of drug doses.

## 7. Challenges of Potential Clinical Development of MERTK Inhibitors

While MERTK targeted therapies show promise, challenges remain. Inhibiting MERTK could lead to upregulation of other TAM family members and promote resistance. Targeting multiple TAM receptors or combination with other targeted therapies or chemotherapies might yield greater efficacy in some therapeutic approaches.

Identifying potential biomarkers which could predict sensitivity to MERTK inhibitor is a major challenge. Biomarkers predicting response to MERTK inhibition could help with patient selection and monitoring thus would greatly improve MERTK inhibitor use.

## 8. Conclusions

This review has aimed to provide an examination of the evolution of our understanding of MERTK from gene discovery to development of inhibitors in clinical trials. We have discussed the historical context of its discovery, signaling pathways that promotes oncogenesis, and possible oncogenic functions in cancer cell nucleus. We have also dissected the role of MERTK in immune system physiology and pathology and provided insight into the interplay of MERTK in cancer and immune microenvironment. MERTK represents a multifaceted target in cancer therapy as MERTK inhibition can impact tumor cells directly as well as modulation of the immune system. Further combination therapies with chemotherapeutic agents or other immune therapies have the potential to enhance treatment outcomes. The clinical trials of MERTK targeted kinase inhibitors are still in early phases of evaluation but in general the clinical agents have been well tolerated with minimal adverse side effects noted, positioning these agents for further study. Moving forward, strategies such as the development of more selective MERTK inhibitors, there will be a focus on use of combination therapies to mitigate resistance and effective biomarkers to predict patient responsiveness. Ongoing and future clinical studies will continue to assess efficacy relative to the safety profile of these therapies.

## Figures and Tables

**Table 1:** MERTK targeting agents in preclinical development.

Therapeutic Modality	Agent Name	Primary Targets	Other Targets	Cancers Targeted
Monoclonal antibody	Mer590	MERTK		NSCLC [70], GBM [47]
	Anti-MerTK antibody	MERTK		TNBC [71], Colorectal cancer [71]
	Anti-Mertk3	MERTK		TNBC [34]
	RGX-019	MERTK		Melanoma [72]
Antibody–drug conjugate	RGX-019-MMAE	MERTK		AML [73,74], TNBC [73], MM [73]
Decoy receptor	MERTK-Fc	MERTK, AXL, TYRO3		Not reported
Targeted protein degrader	KTX-335	MERTK		Not reported
	KTX-652	MERTK, AXL		Breast cancer [75]
	KTX-978	MERTK, AXL, TYRO3		Breast cancer [75], Colon cancer [75]
Ligand–receptor interaction inhibitor	RU-301	AXL, MERTK, TYRO3		TNBC [76], NSCLC [76]
	RU-302	AXL, MERTK, TYRO3		TNBC [76], NSCLC [76]
Small-molecule kinase inhibitor	UNC569	MERTK	AXL, TYRO3, FLT3, MAPKAPK2, RET	AML [77], ALL [78,79], Atypical teratoid/rhabdoid tumors [78,79]
	UNC2025	MERTK	AXL, TYRO3, TRKA, TRKC	AML [80], ALL [80], GBM [81,82], Melanoma [83], NSCLC [51,84], DSRCT [85], HNSCC [84], TNBC [84], Gastric cancer [86]
	CT413	MERTK, AXL, MET, RON		NSCLC [87], Ovarian cancer [87], CML [87]
	LDC1267	MERTK, AXL, TYRO3, MET, Aurora B	LCK, SRC	Breast cancer [88], Melanoma [88]
	SGI-7079	MERTK, FLT3, TRKB, RET, TRKA, YES, AXL, MET, JAK2, KDR, JNK3, ABL	TAK1, SYK, RSK1, CHK2, EPHA1, LCK, GSK3β, EPHB1, FYN, FGFR1	Breast cancer [89], NSCLC [90]

ALL, acute lymphoid leukemia; AML, acute myeloid leukemia; CML, chronic myeloid leukemia; DSRCT, desmoplastic small round cell tumor; GBM, glioblastoma multiforme; HNSCC, head and neck squamous cell carcinoma; MM, multiple myeloma; NSCLC, non-small-cell lung cancer; TNBC, triple-negative breast cancer.

## Supplementary Materials

The following supporting information can be downloaded at: https://www.mdpi.com/article/10.3390/ijms25147660/s1.
