# Supplementary material for: MERTK Inhibition as a Targeted Novel Cancer Therapy
Source: Int J Mol Sci. 2024 Jul 12;25(14):7660. doi: 10.3390/ijms25147660 (PMC11277220; doi:10.3390/ijms25147660)
Supplement: Supplementary file 1 [file ijms-25-07660-s001.zip › ijms-3066412-supplementary.pdf]

Table S1. MERTK targeting agents in clinical development.

Agent name	Therapeutic modality	Targets	Interventions	Malignancies targeted	Development phase	Trial ID ; Status
MRX-2843	Small-molecule kinase inhibitor	MERTK, FLT3, AXL, TRKA, LOK	Monotherapy	Advanced solid tumors	Phase I	NCT03510104 ; Completed
			Monotherapy	R/R AML, ALL, or MPAL	Phase I	NCT04872478 ; Recruiting
			In combination with osimertinib (EGFR TKI)	EGFR-mutant NSCLC	Phase I	NCT04762199 ; Recruiting
ONO-7475	Small-molecule kinase inhibitor	MERTK, AXL	Monotherapy or in combination with nivolumab (anti-PD-1 antibody)	Advanced or metastatic solid tumors	Phase I	NCT03730337 ; Completed
Q702	Small-molecule kinase inhibitor	AXL, MERTK, CSF1R	Monotherapy	Advanced solid tumors	Phase I	NCT04648254 ; Recruiting
			In combination with pembrolizumab (anti-PD1 antibody)	Advanced esophageal, gastric/GEJ, hepatocellular, and cervical cancers	Phase I/II	NCT05438420 ; Recruiting
			In combination with azacitidine and venetoclax	R/R AML	Phase I	NCT06445907 ; Not yet recruiting
PF-07265807	Small-molecule kinase inhibitor	AXL, MERTK	Monotherapy	Advanced or metastatic solid tumors	Phase I	NCT04458259 ; Active, not recruiting
			In combination with sasanlimab (anti-PD-1 antibody)			
			In combination with sasanlimab and axitinib (VEGFR TKI)			
INCB081776	Small-molecule kinase inhibitor	AXL, MERTK, MET	Monotherapy or in combination with retifanlimab (anti-PD-1 antibody)	Advanced or metastatic solid tumors , R/R AML	Phase I	NCT03522142 ; Active, not recruiting
			In combination with pembrolizumab (anti-PD-1 antibody) and palliative radiation therapy	Recurrent/metastatic HNSCC	Phase I	NCT06308913 ; Recruiting
RXDX-106	Small-molecule kinase inhibitor	AXL,TYRO3, MET, MERTK, RON, ABL1, LCK, TRKA/C	Monotherapy	Advanced or metastatic solid tumors	Phase I	NCT03454243 ; Terminated
BMS-777607 (ASLAN002)	Small-molecule kinase inhibitor	AXL, RON, MET, TYRO3, MERTK, FLT3, AURKB, LOK, TIE2, MKNK2, MUSK	Monotherapy	Advanced or metastatic solid tumors	Phase I/II	NCT00605618 ; Completed
			Monotherapy	Advanced or metastatic solid tumors	Phase I	NCT01721148 ; Completed
XL092	Small-molecule kinase inhibitor	MET, VEGFR2, MER, AXL	In combination with atezolizumab (anti-PD-L1 antibody)	Metastatic colorectal cancer	Phase III	NCT05425940 ; Recruiting
			In combination with nivolumab (anti-PD-1 antibody)	Advanced or metastatic nccRCC	Phase III	NCT05678673 ; Recruiting
			Monotherapy or in combination with atezolizumab (anti-PD-L1 antibody) or in combination with avelumab (anti-PD-L1 antibody)	Advanced solid tumors	Phase I	NCT03845166 ; Active, not recruiting
			In combination with nivolumab (anti-PD-1 antibody) or in combination with ipilimumab (anti-CTLA4 antibody) and nivolumab or in combination with nivolumab + relatlimab (anti-LAG-3 antibody)	Advanced solid tumors	Phase I	NCT05176483 ; Recruiting
			In combination with AB521(HIF-2α inhibitor) or in combination with AB521 and nivolumab (anti-PD-1 antibody)	Advanced ccRCC or other advanced solid tumors	Phase I	NCT06191796; Recruiting
			In combination with pembrolizumab (anti-PD-1 antibody)	PD-L1 positive recurrent or metastatic HNSCC	Phase II/III	NCT06082167; Recruiting
			In combination with atezolizumab (anti-PD-L1 antibody) and tiragolumab (anti-TIGIT antibody)	Metastatic NSCLC	Phase I/II	NCT03337698 ; Active, not recruiting
Merestinib (LY2801653)	Small-molecule kinase inhibitor	MERTK, DDR1, AXL, MKNK1/2, FLT3, DDR2, RON, ROS1, TYRO3, MET, PDGFRA, TEK	In combination with LY2874455 (FGFR TKI)	R/R AML	Phase I	NCT03125239 ; Completed
			Monotherapy or in combination with cisplatin and gemcitabine	Advanced and/or metastatic solid tumors or non-Hodgkin's lymphoma	Phase I	NCT03027284 ; Completed
			In combination with cisplatin and gemcitabine	Advanced or metastatic biliary tract cancer	Phase II	NCT02711553 ; Active, not recruiting
			In combination with ramucirumab (anti-VEGFR-2 antibody)	Advanced or metastatic colorectal cancer, MCL	Phase I	NCT02745769 ; Completed
			Monotherapy or in combination with cetuximab (anti-EGFR antibody) or in combination with cisplatin or in combination with cisplatin and gemcitabine or in combination with ramucirumab (anti-VEGFR-2 antibody)	Advanced cancer	Phase I	NCT01285037; Completed
			In combination with LY3300054 (anti-PD-L1 antibody)	Pancreatic cancer	Phase I	NCT02791334 ; Active, not recruiting
Ningetinib (CT053PTSA)	Small-molecule kinase inhibitor	c-Met, VEGFR2, AXL, MERTK, and FLT3	Monotherapy	Advanced NSCLC	Phase II	NCT04992858 ; Not yet recruiting
			Monotherapy	Advanced solid tumors	Phase I	NCT04577703 ; Completed
AT9283	Small-molecule kinase inhibitor	AURKA/B, JAK2/3, ABL1, MERTK, GSK3β, FGFR2, VEGFR3, RET, RSK2/3, TYK2, YES, FLT3, DRAK1, FGFR1/3, VEGFR1/2, PDGFRA, PDK1	Monotherapy	Multiple myeloma	Phase II	NCT01145989 ; Completed
			Monotherapy	Advanced or metastatic solid tumors or non-Hodgkin's lymphoma	Phase I	NCT00443976 ; Completed
			Monotherapy	R/R acute leukemias	Phase I	NCT01431664 ; Completed
			Monotherapy	R/R solid tumors	Phase I	NCT00985868 ; Completed
Foretinib	Small-molecule kinase inhibitor	AXL, MET, MERTK, VEGFR2/3, TIE2, TYRO3, RON, FLT3, PDGFRA, VEGFR1, KIT, PDGFRB, ROS1	Monotherapy	Hepatocellular carcinoma	Phase I	NCT00920192 ; Completed
			Monotherapy	Breast cancer	Phase II	NCT01147484 ; Completed
			In combination with lapatinib (EGFR/HER2 TKI)	Breast cancer	Phase I/II	NCT01138384 ; Completed
			Monotherapy	Solid tumors	Phase I	NCT00742131 ; Completed
			Monotherapy	Head and neck cancer	Phase II	NCT00725764 ; Completed
			Monotherapy	Papillary renal cell carcinoma	Phase II	NCT00726323 ; Completed
			Monotherapy	Solid tumors	Phase I	NCT00742261 ; Completed
			Monotherapy	Metastatic gastric cancer	Phase II	NCT00725712 ; Completed
			Monotherapy	Solid tumors	Phase I	NCT00743067 ; Completed
			In combination with erlotinib (EGFR TKI)	Advanced or metastatic NSCLC	Phase I/II	NCT01068587 ; Completed
S49076	Small-molecule kinase inhibitor	MET, AXL, FGFR1/2/3	Monotherapy	Advanced solid tumors	Phase I	ISRCTN00759419; Completed
			In combination with bevacizumab (anti-VEGF antibody)	GBM	Phase I/II	EudraCT2013-003079-37 or ISRCTN11619481; Completed
			In combination with gefitinib (EGFR TKI)	NSCLC	Phase I/II	EudraCT2015-002646-31 ; Completed
Sitravatinib	Small-molecule kinase inhibitor	DDR2, EPHA3, AXL, MERTK, TYRO3, FLT3, VEGFR, PDGFR, KIT, MET, RET, TRKA, TRKB	Monotherapy	Advanced liposarcoma and other soft tissue sarcomas	Phase II	NCT02978859 ; Active, not recruiting
			In combination with tislelizumab (anti-PD-1 antibody)	HCC	Phase II	NCT05407519 ; Recruiting
			In combination with tislelizumab (anti-PD-1 antibody)	Advanced biliary tract cancer	Phase II	NCT04727996; Active, not recruiting
			In combination with nivolumab (anti-PD-1 antibody)	Metastatic or advanced ccRCC	Phase II	NCT04904302; Active, not recruiting
			In combination with tislelizumab (anti-PD-1 antibody)	ES-SCLC	Phase II	NCT05228496; Active, not recruiting
			In combination with tislelizumab (anti-PD-1 antibody) or in combination with tislelizumab and nab-paclitaxel	Recurrent or metastatic TNBC	Phase II	NCT04734262; Active, not recruiting
			In combination with tislelizumab (anti-PD-1 antibody)	Metastatic uveal melanoma	Phase II	NCT05542342; Active, not recruiting
			Monotherapy	NSCLC	Phase II	NCT02664935; Active, not recruiting
			In combination with nivolumab (anti-PD-1 antibody)	Advanced non-squamous NSCLC	Phase III	NCT03906071; Active, not recruiting
			Monotherapy or in combination with other anitcancer therapies	Advanced or metastatic solid malignancies	Phase II/III (as extension of parent clinical trial)	NCT04887870 (Extension trial) ; Active, not recruting
			Monotherapy or in combination with tislelizumab (anti-PD-1 antibody)	Advanced malignancies	Phase III (as extension of parent clinical trial)	NCT04164199 (Extenstion trial) ; Enrolling by invitation
SLC-391	Small-molecule kinase inhibitor	AXL, FGFR3, VEGFR, MERTK, TYRO3, FGFR2, ALK	Monotherapy	Solid tumors	Phase I	NCT03990454 ; Completed
			In combination with pembrolizumab (anti-PD1 antibody)	Advanced or metastatic NSCLC	Phase I/II	NCT05860296 ; Recruiting

For each agent, all active or completed (non-terminated) clinical trails in cancer patients have been included except for S49076, sitravatinib, and RXDX-106. Listed trials are the only known trials for RXDX-106. As S49076 clinical trails are not listed in www.clinicaltrials.gov, a more comprehensive trial list for this agent could not be formulated. For sitravatinib, only active trails are included in the interest of brevity. For a more comprehensive list visit : www.clinicaltrials.gov

ALL, acute lymphoid leukemia; AML, acute myeloid leukemia; ccRCC, clear cell renal cell carcinoma; CML, chronic myeloid leukemia; ES-SCLC, extensive stage small cell lung cancer; GBM, glioblastoma multiforme; GEJ, gastroesophageal junction; HNSCC, head and neck squamous cell carcinoma; MCL, mantle cell lymphoma ; MDS, myelodysplastic syndrome; MPAL, mixed-phenotype acute leukemia; nccRCC, non-clear cell renal cell carcinoma; NSCLC, non-small-cell lung cancer; R/R, relapsed refractory; TNBC, triple negative breast cancer; TKI, tyrosine kinase inhibitor
